# Reach, engagement and effectiveness of in-person and online lifestyle change programs to prevent diabetes

**DOI:** 10.1186/s12889-021-11378-4

**Published:** 2021-07-05

**Authors:** Ilya Golovaty, Sandeep Wadhwa, Lois Fisher, Iryna Lobach, Byron Crowe, Ronli Levi, Hilary Seligman

**Affiliations:** 1 Division of General Internal Medicine, University of Washington School of Medicine, Seattle, WA USA; 2 Division of Geriatric Medicine, University of Colorado School of Medicine, Aurora, CO USA; 3 3M Health Information Systems Division, Murray, UT USA; 4 Division of General Internal Medicine, Department of Medicine, University of California San Francisco, San Francisco, CA USA; 5 UCSF Center for Vulnerable Populations at Zuckerberg San Francisco General Hospital, San Francisco, CA USA; 6 Department of Epidemiology and Biostatistics, University of California San Francisco, San Francisco, CA USA; 7 Solera Health, Phoenix, AZ USA

**Keywords:** Diabetes prevention programs, Digital, Virtual, DPP, Lifestyle change programs, LCP

## Abstract

**Background:**

COVID-19 has accelerated interest in and need for online delivery of healthcare. We examined the reach, engagement and effectiveness of online delivery of lifestyle change programs (LCP) modelled after the Diabetes Prevention Program (DPP) in a multistate, real-world setting.

**Methods:**

Longitudinal, non-randomized study comparing online and in-person LCP in a large multistate sample delivered over 1 year. Sample included at-risk adults (*n* = 26,743) referred to online (*n* = 9) and in-person (*n* = 11) CDC-recognized LCP from a multi-state registry (California, Florida and Colorado) between 2015 and 2018. The main outcome was effectiveness (proportion achieving > 5% weight loss) at one-year. Our secondary outcomes included reach (proportion enrolled among referred) and engagement (proportion ≥ 9 sessions by week 26). We used logistic regression modelling to assess the association between participant- and setting -level characteristics with meaningful weight loss.

**Results:**

Online LCP effectiveness was lower, with 23% of online participants achieving > 5% weight loss, compared with 35% of in-person participants (*p* < 0.001). More adults referred to online programs enrolled (56% vs 51%, *p* < 0.001), but fewer engaged at 6-months (attendance at ≥9 sessions 46% vs 66%, *p* < 0.001) compared to in-person participants.

**Conclusions:**

Compared to adults referred to in-person LCP, those referred to online LCP were more likely to enroll and less likely to engage. Online participants achieved modest meaningful weight loss. Online delivery of LCP is an attractive strategy to deliver and scale DPP, particularly with social distancing measures currently in place. However, it is unclear how to optimize delivery models for maximal impact given trade-offs in reach and effectiveness.

**Supplementary Information:**

The online version contains supplementary material available at 10.1186/s12889-021-11378-4.

## Background

Nearly one in three adults in the United States (US) have prediabetes, with an annual incident risk of type 2 diabetes (T2D) around 5% without intervention [1]. Structured lifestyle change programs (LCP^1^), based on the Diabetes Prevention Program (DPP) trials, promote behavioral change to improve diet, increase physical activity and manage weight [2]. These lifestyle modifications work in tandem to achieve weight loss and may reduce T2D incident risk by up to half, making it one of the most effective ways available to prevent progression to T2D [3]. Numerous LCP-related trials have demonstrated clinically meaningful weight loss in real-world settings [4–6]. However, only 4 % of the estimated 84 million at-risk adults in the US have been referred to a 1 year LCP [7].

Online LCP may be a highly scalable approach to address the low referral rates of at-risk adults [8]. Currently 76 online LCPs are recognized by CDC [9]. Compared to in-person programs, they may be more accessible, personalized, lower cost and more easily integrated into existing health systems. In addition, the COVID-19 pandemic has rapidly built momentum for implementation of online and other technology-enabled alternatives for delivery of critical healthcare services while maintaining social distance.

Existing studies examining the comparability of online and in-person LCP delivery have been single-site studies [10] and/or smaller studies funded by online LCP providers [11–13]. Studies comparing technology-based vs in-person delivery of similar types of interventions, such as weight loss [14, 15] and diabetes self-management [16] programs, have been inconclusive. Therefore, we sought to examine the real-world delivery of both in-person and online LCP by reach, engagement and effectiveness within a large, multi-state registry.

## Methods

We performed a longitudinal analysis of at-risk adults referred to numerous online and in-person LCP from a multi-state registry to a) assess overall reach and effectiveness of online LCP, and b) identify participant- and setting-level factors related to participant engagement and weight loss.

The registry data was provided by a private platform, Solera Health [17]. Details of the referral registry are provided in the supplemental material (Additional file 1). The research protocol was exempted for review by University of California San Francisco’s Human Research Protection Program.

### Population

The sample included adults referred to LCP using the Solera Health platform between 2015 and 2018. Eligibility criteria included ages 18–85 years and at high risk for diabetes. “High risk” for diabetes was defined as at-risk weight for diabetes (body mass index (BMI) > 24 kg/m^2^ or > 22 kg/m^2^ among Asian American), suggestive blood glucose testing (fasting glucose of 100–125 mg/dl, plasma glucose of 140–199 mg/dl measured 2 h after a 75 g glucose load, or glycosylated hemoglobin of 5.7–6.4), history of gestational diabetes, or high-risk on a self-administered CDC written prediabetes risk assessment [18]. Adults with government-sponsored insurance or no insurance were ineligible. Eligible participants registered for an online account and completed health and demographic questions. Participants whose address was within 25 miles of an in-person program with open enrollment were given a choice of in-person or online LCP; this subset of participants was not identifiable within the dataset. If in-person programs were unavailable, only an online program was offered.

**Fig. 1:**
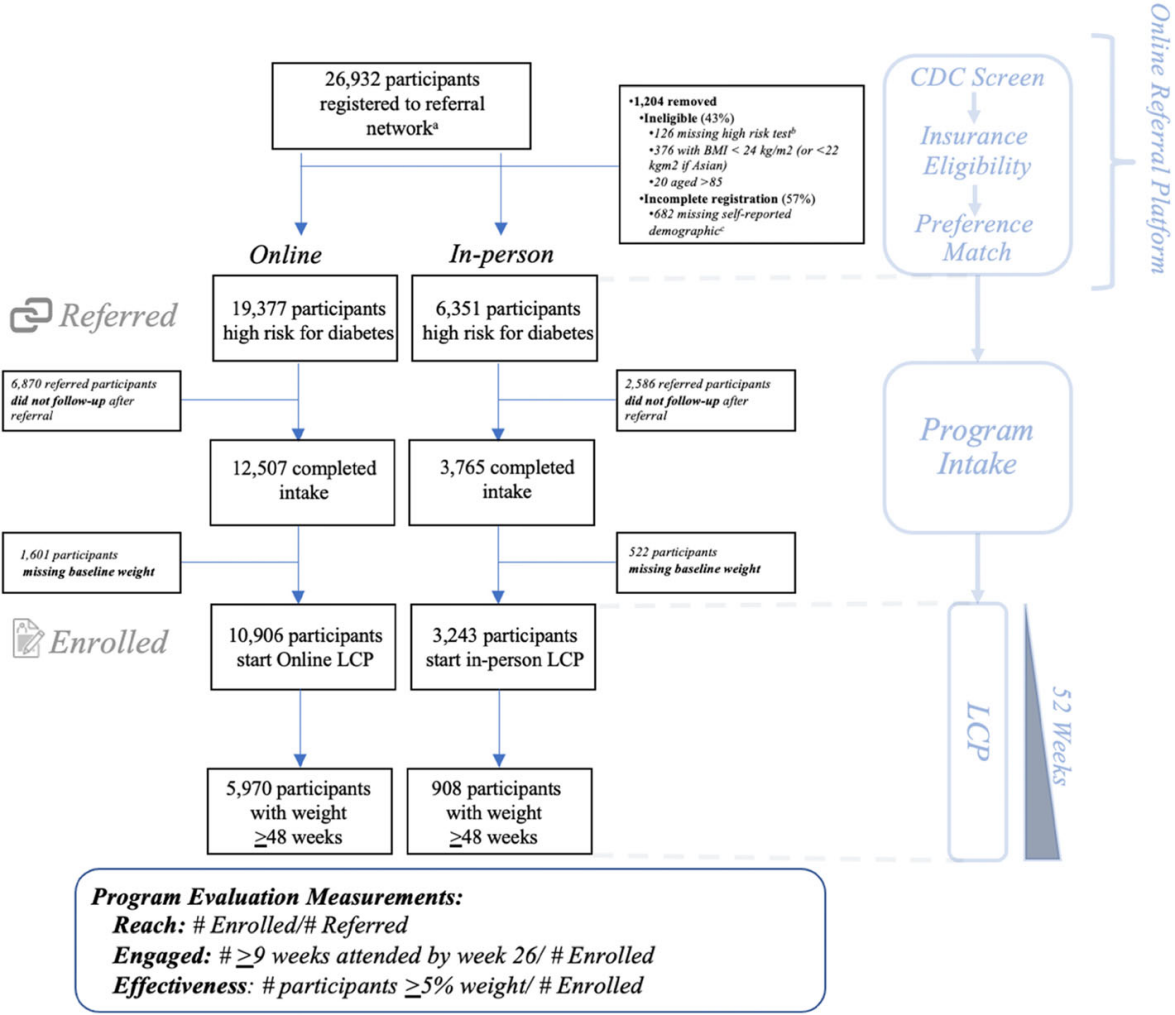
Participant Flowchart in a Multistate Referral Registry to Community Lifestyle Change Programs to Prevent Diabetes (2015–2018). ^a^Analysis includes 26,743 unique participants. One thousand three hundred twenty-eight surplus registrations records were removed (e.g. participant had ≥1 registration record). One hundred sixty-five individuals participated in multiple programs (157 participants in two programs, 8 participants in three programs) during the study period and were included in the analysis; among these individuals, 82 participated in both an in-person and online program and were included in both arms of the analysis. ^b^Fasting glucose of 100 to 125 mg/dl, plasma glucose of 140 to 199 mg/dl measured 2 h after a 75 g glucose load, A1c of 5.7 to 6.4, history of gestational diabetes and/or a high-risk on a self-administered CDC prediabetes risk assessment. ^c^Individuals that self-identified as multiracial or did not report race or ethnicity were defined as ‘other’

The full registry included 26,932 registration records from 26,743 unique individuals referred to 20 CDC-recognized LCP (9 online, range of records registered by organization 1–34% of online registrants; and 11 in-person, range of records registered by organization 1–80% of in-person registrants). One thousand two hundred four registration records were excluded for ineligibility (*n* = 518) or incomplete registration (*n* = 686). The final analytic sample included 25,728 registration records (19,377 online LCP, 6351 in-person LCP, see Fig. 1).

### Interventions

Online LCP were defined as year-long programs delivering the entire CDC-approved LCP curriculum online with peer support, self-monitoring of diet and physical activity and virtual interaction with a human coach [18]. Online programs deliver the standardized content asynchronously in varying modules and formats.

In-person LCP were group-coaching programs with at least sixteen weekly core sessions in the first 6 months and bimonthly maintenance sessions through the remainder of the year [2]. All the organizations in this study were pending, preliminary or met full recognition per the CDC standard through the CDC Diabetes Prevention Recognition Program (DPRP) at the time of the study.

### Analysis

Our main implementation outcome was effectiveness, defined as the proportion of enrolled participants achieving > 5% weight loss during the year-long intervention. Weight change was calculated as percentage change in measured weight from baseline weight to the last recorded weight (online: recorded by cellular scales transmitted using cellular data networks from the participants’ home; in-person: weigh-in during weekly session).

Our secondary outcomes included reach and engagement. We selected the secondary outcomes a priori to explore mechanisms leading to our primary endpoint and to evaluate the public health impact of LCP.

Reach was defined as number of participants enrolled in LCP divided by the number of participants referred to LCP. At-risk adults were considered ‘referred’ after they a) registered on the Solera platform, b) qualified for enrollment, and c) matched to a program. Program matching was based on individual preferences (class size, online vs. in-person, class schedule, and location; See Additional file 1). Participants were considered ‘enrolled’ if they completed their intake questionnaire and provided a baseline weight (online: set-up their cellular scale and completed their baseline weight; in-person: baseline weight measured by the health coach at class intake).

In-person engagement was defined as physical attendance at the weekly hour-long session tallied by the health coach. Weekly online engagement was defined by a proprietary algorithm which includes curriculum delivery, health coach interaction, peer support and self-tracking that is equivalent to an hour of in-person class engagement. The CDC standard for engagement during the time of data collection was engagement at ≥9 sessions by week 26, a threshold associated with meaningful weight loss [18]. Six month engagement in this analysis was defined as the number of participants reaching the CDC-defined engagement threshold at 26 weeks divided by the number of participants enrolled. We also examined the adjusted odds of participants achieving meaningful weight loss for every session engaged.

### Covariates

We stratified outcomes by participant-, setting- and intervention-level characteristics. Participant-level variables included demographics (self-reported age, sex, and race/ethnicity), baseline body mass index (BMI; normal < 25 kg/m^2^, overweight 25–29 kg/m^2^ and obese > 30 kg/m^2^) and method of determining elevated diabetes risk (blood glucose testing, history of gestational diabetes, or high-risk on a self-administered CDC written prediabetes risk assessment). Individuals that self-identified as multiracial or did not report race or ethnicity were defined as ‘other’. Setting-level variables included median income of zip code of residence and rural/urban designation. Income estimates were generated using participant ZIP codes cross-tabulated to ZIP Code Tabulation Areas (ZCTAs) created by the US Census Bureau [19]. Median household income estimates were generated based on ZCTA-level estimates from the 2016 American Community Survey [20]. Rurality estimates were generated by cross-tabulating participant ZIP codes to Rural–Urban Commuting Area Codes (RUCA) [21]. Intervention-level characteristics included registration date and LCP provider.

### Statistical analysis

We used t-tests for continuous, normally distributed variables, non-parametric testing for non-normally distributed variables, and χ^2^ tests for categorical variables. We used logistic regression modelling to assess the association between participant- and setting-level characteristics with effectiveness (primary outcome), reach and engagement. We used multilevel mixed-effects modelling with participants nested within the program as random effect, adjusted for participant- and setting-level factors, to estimate per week percent weight loss. We used separate models for in-person and online programs to account for varying patterns in weekly program missingness (higher missingness in weekly weight measures among in-person participants – see Additional file 2). A *p*-value of < 0.05 was considered to be statistically significant. All analyses were conducted using Stata 15.0 (Stata Corporation, College Station, TX, USA).

### Sensitivity analyses

We examined effectiveness among participants who engaged in at least four sessions, the minimal exposure defined by the CDC as engagement at the time of program participation [18]. We opted to examine this subset of the population as a sensitivity analysis, rather than our primary population, to minimize potential measurement error from the varying definitions of program engagement between online and in-person platforms.

The comparative ease of home weigh-ins may have resulted in differentially more last recorded weigh-ins among less engaged online participants who regained weight and remained in their program compared to in-person participants who may have stopped attending their program after regaining weight. Therefore, we also investigated effectiveness defined as percentage weight change from baseline weight to the lowest recorded weight.

Lastly, we excluded participants who participated in multiple programs to limit the effect of individuals with higher propensity to engage (*n* = 165 participants, see Fig. 1 footnote for details).

## Results

### Baseline characteristics of adults referred to a LCP

Table 1 displays characteristics of at-risk adults referred to LCP by enrollment status. Overall, participants referred to LCP were mostly female (75% female), non-Hispanic (61%), White (56%) and from urban areas (85%). Participants were considered referred after they registered and matched to a lifestyle change program per their preferences. Participants that lived more than 25 miles from the closest in-person program were limited to online programs. Participants matched to online LCP were mostly non-Hispanic (64%), White, from California or Florida (59 and 27%, respectfully), and living in higher-income, urban-designated areas. Compared to adults who were matched to in-person programs, adults matched to online LCP were significantly younger (median age 50 vs 52,*p* < 0.001), male (28% vs 16%,*p* < 0.001), more white (White 58% vs 51%,*p* < 0.001) and lived in more rural areas (10% vs 5%,*p* < 0.001). A lower percentage of adults who enrolled in an online program were obese compared with adults enrolled in an in-person program (65% vs 73%,*p* < 0.001).

**Table 1:** Characteristics of High-risk Adults Referred to a Community Lifestyle Change Program to Prevent Diabetes in a Multistate Referral Registry by Delivery Type (2015–2018)

	Overall	Online				In-Person			
	Referred (*n* = 25,728)	Referred^a^ (*n* = 19,377)	Enrolled^b^ (*n* = 10,906)	Not Enrolled^ (*n* = 8471)	*p* value^c^,	Referred^a^ (*n* = 6351)	Enrolled^b^ (*n* = 3243)	Not Enrolled (*n* = 3108)	*p* value^c^,
**Participant**									
Age (median, IQR)	51 (41–59)	50 (40–58)	49 (39–57)	52 (42–60)	< 0.001	52 (42–60)	53 (43–60)	52 (42–60)	0.03
Sex (% male)	25	28	26	31	< 0.001	16	11	20	< 0.001
Race (%)									
African American	6	6	6	5	< 0.001	8	7	8	< 0.001
White	56	58	59	56		51	54	48	
Asian American	3	4	3	4		3	1	4	
Native American	1	1	1	1		1	0	1	
Other^d^	34	32	31	34		37	37	39	
Ethnicity									
Hispanic	15	14	14	13	0.002	18	15	21	< 0.001
Non-Hispanic	61	64	65	63		53	51	55	
Not reported	24	23	22	24		30	35	25	
**Setting**									
Region (%)									
California	59	59	57	63	< 0.001	59	53	65	< 0.001
Florida	29	27	29	28		28	30	26	
Colorado	5	4	5	3		10	14	6	
Other	7	8	9	7		2	3	2	
Household Income^e^									
< 40,000	13	13	13	13	0.001	12	12	12	0.11
40,001-50,000	21	21	21	20		20	21	20	
50,001-63,000	25	25	25	24		26	26	25	
> 63,000	42	42	40	43		42	41	44	
Rurality^f^									
Urban	85	84	83	85	0.002	90	88	91	< 0.001
Suburban	6	6	7	6		5	6	5	
Rural	9	10	11	10		5	6	4	
**Clinical**									
Baseline BMI									
Normal (< 25 kg/m^2^)	–	–	3	–	–	–	2	–	–
Overweight (25–29 kg/m^2^)	–	–	32	–		–	26	–	
Obese (> 30 kg/m^2^)	–	–	65	–		–	73	–	
Screening Type (%)									
Blood Glucose Screen^g^	30	29	30	27	< 0.001	33	38	29	< 0.001
Gestational Diabetes	1	1	1	1	0.22	2	2	1	< 0.001

*BMI* body mass index, *IQR* Interquartile range

^a^Participants were considered referred after they registered and matched to a lifestyle change program per their preferences. Participants that lived more than 25 miles from closest in-person program were limited to online programs

^b^Defined as high-risk adults who completed registration and collected baseline weight

^c^*p* values estimating proportion ‘Enrolled’ vs ‘Not Enrolled’ using chi-2 tests for categorical variables, t-test for normally distributed and Wilcoxon tests for non-parametric continuous variables

^d^Individuals that self-identified as multiracial or did not report race were defined as ‘other’. Hawaiian/PI < 1%

^e^Household income estimates based on participant zip codes using Zip Code Tabulation Area median household incomes from the US Census Data

^f^Rurality estimates based on participant zip codes using the Rural–Urban Commuting Area Codes (RUCA) which uses 2010 census data on the basis of population density, urbanization, and daily commuting patterns

^g^Fasting glucose of 100 to 125 mg/dl, plasma glucose measured 2 h after a 75 g glucose load of 140 to 199 mg/dl, or A1c of 5.7 to 6.4

### Effectiveness

**Fig. 2:**
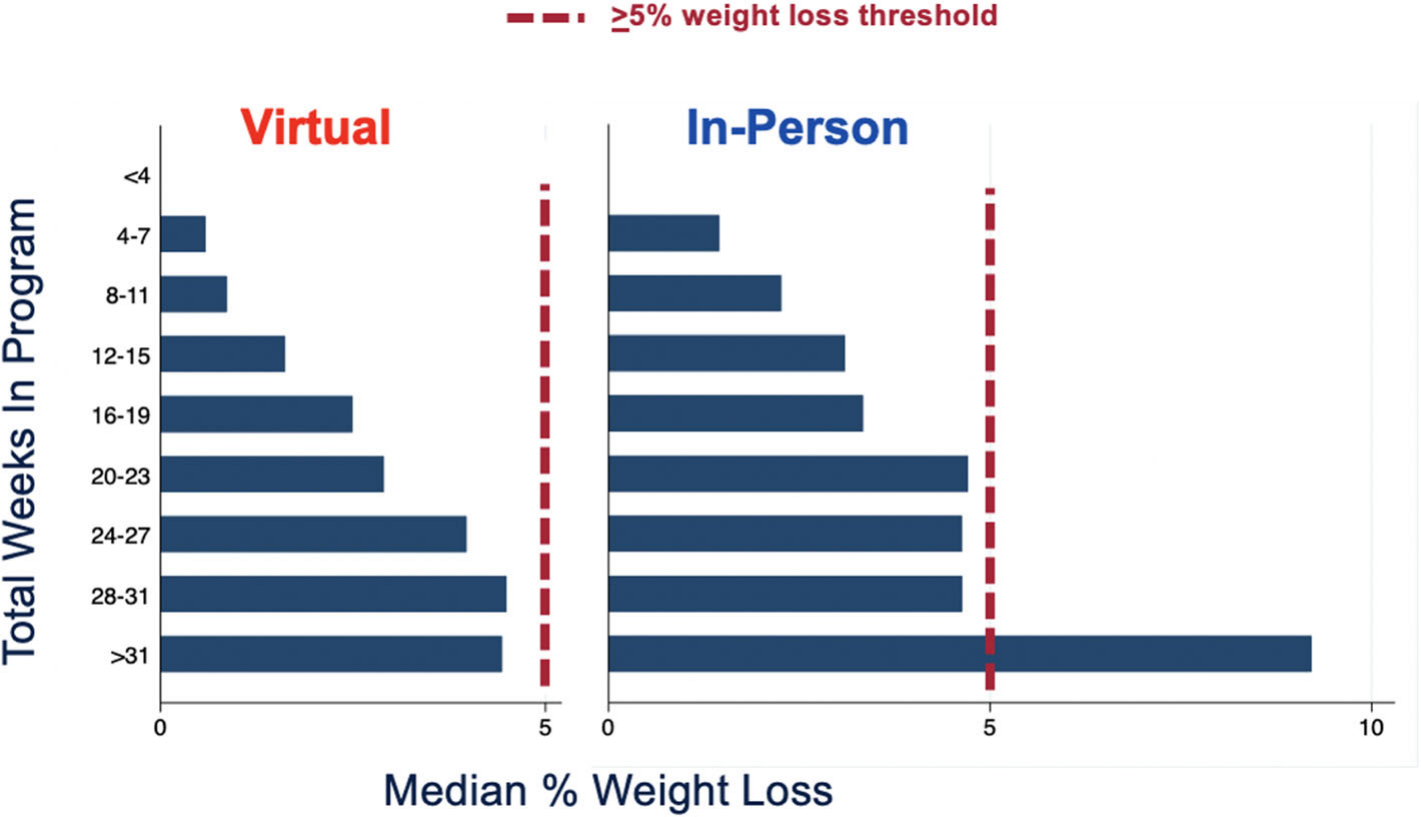
Median Weight Loss by Total Program Week Engaged^a^ among Adults Enrolled in a Lifestyle Change Programs to Prevent Diabetes (2015–2018). ^a^Each online program defined engagement as a composite of subcomponents equivalent to an in-person hour session. Subcomponents included curriculum delivery, health coach interaction, peer support and self-tracking (physical and/or dietary). Each program had a unique threshold for engagement deemed equivalent to an hour of in-person class, defined between the program and commercial insurer for reimbursement

Table 2 shows the descriptive findings of the main implementation outcomes. Online LCP effectiveness was lower, with 23% of online-enrolled participants achieving the 5% weight loss threshold, compared with 35% of in-person-enrolled participants (*p* < 0.001). Meaningful weight loss differed significantly by specific online provider in this study (16–27%,*p* < 0.001; data not shown). Mean weight loss was 2.0% (95%CI 1.9–2.1) among online participants compared to 4.3% (95%CI 4.1–4.5) among in-person participants. Median percent weight loss increased by total program week engagement but was consistently below the meaningful weight loss threshold among participants enrolled in online programs (Fig. 2). Table 3 presents the odds ratios of effectiveness among those enrolled by participant- and setting-level factors. After adjusting for age, sex, race/ethnicity, baseline BMI, region, income, rural/urban, registration date and program, the odds of online participants achieving meaningful weight loss was lower among those 18–44 years old (AOR 0.67,95%CI 0.56–0.80) and 45–64 years old (AOR 0.80,95%CI 0.68–0.94) than among those ages 65 and older (Table 3). Similarly, meaningful weight loss among online participants was lower among African American participants compared to White participants (*p* < 0.05). Multilevel modelling revealed that adjusted weight loss was 0.07% per program week among online programs and 0.11% per program week among in-person programs.

**Table 2:** Implementation Outcomes in Community Lifestyle Change Program to Prevent Diabetes in a Multistate Referral Registry (2015–2018)

Implementation Measures	n (%)		*P* value^a^
	Online	In-Person	
Referred ^b^	(*n* = 19,377)	(*n* = 6351)	
**Reach**			
*(Enrolled/Referred)*	10,906 (56%)	3243 (51%)	*p* < 0.001
Enrolled	(*n* = 10,906)	(*n* = 3243)	
**Engagement**			
*(>* *9 weeks attended* ^c^ *by week 26 / Enrolled)*	4973 (46%)	2130 (66%)	*p* < 0.001
**Effectiveness**^d,e^			
*(>* *5% weight loss/Enrolled)*	2524 (23%)	1123 (35%)	*p* < 0.001
*Mean weight loss among Enrolled*	2.0% (95%CI 1.9,2.1)	4.3% (95%CI 4.1, 4.5)	
*Median weight loss among Enrolled*	1.0% (IQR 0, 4.6)	2.6% (IQR 0, 7.0)	

^a^*p* values estimating proportion achieving implementation outcome using chi-2 tests

^b^Participants were considered referred after they registered and matched to a lifestyle change program per their preferences. Participants that lived more than 25 miles from closest in-person program were limited to online programs

^c^Attendance measured by platform: online: composite of curriculum delivery, health coach interaction, peer support and self-tracking that is measured equivalent of in-person hour attendance and agreed upon between the payer and program; In-person: physical attendance of hour long weekly session

^d^% weight loss calculated by % change from baseline to last recorded weight

^e^Unadjusted mean loss among participants enrolled AND > 4 weeks attended

(In-person *n* = 2762; Online *n* = 7591)

Online: 28% reached > 5%; mean 2.6% (95%CI 2.5, 2.8); median 1.8 (IQR + 0.1, 5.6)

In-person: 41% reached > 5%; mean 5.0% (95%CI 4.7, 5.2); median 3.6 (IQR 0.1, 8.1)

**Table 3:** Adjusted Odd Ratios of Reach, Engagement and Effectiveness by Participant- and Setting-level characteristics in Community Lifestyle Change Programs to Prevent Diabetes in a Multistate Referral Registry (2015–2018)

	Reach Enrolled/ Referred		Engagement > 9 weeks Engaged^d^ by Week 26 / Enrolled		Effectiveness > 5% weight loss/Enrolled	
	Online *n* = 10,906/19,377 AOR^a^ (95%CI)	In-Person *n* = 3243/6351 AOR^a^ (95%CI)	Online *n* = 4973/10,906 AOR^b^ (95%CI)	In-Person *n* = 2130/3243 AOR^b^ (95%CI)	Online *n* = 2524/10,906 AOR^c^ (95%CI)	In-Person *n* = 1123/3243 AOR^c^ (95%CI)
**Participant**						
Age (ref > 65)						
18-44	2.02 (1.81–2.25)***	0.94 (0.78–1.13)	0.39 (0.33–0.45)***	0.38 (0.29–0.50)***	0.67 (0.56–0.80)***	0.74 (0.55–0.98)*
45–64	1.55 (1.40–1.71)***	1.04 (0.89–1.23)	0.72 (0.62–0.84)***	0.75 (0.58–0.97)*	0.80 (0.68–0.94)**	0.66 (0.51–0.84)**
Sex (ref male)	1.24 (1.16–1.32)***	1.77 (1.52–2.05)***	1.18 (1.08–1.29)*	1.03 (0.82–1.31)	0.95 (0.86–1.06)	0.63 (0.49–0.81)***
Race (ref white)						
African American	0.97 (0.85–1.11)	0.71 (0.57–0.87)***	0.95 (0.80–1.13)	0.47 (0.35–0.65)***	0.80 (0.65–0.99)*	0.60 (0.41–0.87)**
Asian American	0.78 (0.64–0.88)**	0.46 (0.32–0.66)***	0.90 (0.71–1.16)	0.65 (0.35–1.21)	0.93 (0.71–1.22)	0.47 (0.21–1.08)
Native American	0.86 (0.62–1.19)	0.70 (0.26–1.85)	0.81 (0.51–1.26)	1.30 (0.35–4.89)	0.69 (0.39–1.23)	1.08 (0.32–3.65)
Other^e^	0.80 (0.72–0.87)***	0.79 (0.67–0.93)**	0.90 (0.80–1.02)	0.96 (0.77–1.21)	0.90 (− 0.77–1.04)	1.02 (0.80–1.31)
Ethnicity (ref non-Latino)						
Latino	1.08 (0.95–1.22)	0.90 (0.74–1.10)	0.87 (0.74–1.02)	0.68 (0.51–0.91)	0.92 (0.76–1.11)	0.86 (0.63–1.18)
BMI (ref Overweight)	–	–				
Obese	–	–	0.88 (0.80–0.95)**	1.06 (0.89–1.26)	0.99 (0.90–1.10)	1.09 (0.91–1.31)
**Setting**						
Region (Ref California)						
Florida	1.10 (1.01–1.19)*	1.31 (1.15–1.49)***	0.92 (0.83–1.02)	0.86 (0.71–1.03)	1.07 (.091–1.25)	1.17 (0.85–1.49)
Colorado	2.06 (1.67–2.54)***	1.62 (1.27–2.06)***	1.09 (0.86–1.39)	1.20 (0.89–1.62)	0.79 (0.58–1.07)	0.93 (0.75–1.17)
Other	1.22 (1.08–1.36)**	1.19 (0.85–1.67)	1.06 (0.92–1.23)	0.86 (0.53–1.38)	1.18 (0.99–1.40)	0.90 (0.74–1.11)
Income (ref: high quartile)^f^						
< 40,000	0.93 (0.84–1.03)	0.96 (0.80–1.15)	0.98 (0.86–1.12)	1.05 (0.81–1.36)	1.07 (0.91–1.25)	1.13 (0.85–1.49)
40,001-50,000	1.02 (0.94–1.11)	0.99 (0.85–1.14)	0.94 (0.85–1.06)	1.04 (0.84–1.28)	1.04 (0.92–1.19)	0.93 (0.75–1.17)
50,001-63,000	1.02 (0.94–1.10)	0.97 (0.85–1.11)	0.96 (0.87–1.07)	0.96 (0.80–1.16)	0.96 (0.85–1.09)	0.90 (0.74–1.11)
Rurality (ref Urban)^g^						
Suburban	1.10 (0.97–1.24)	1.01 (0.80–1.26)	1.16 (0.99–1.36)	0.95 (0.69–1.32)	1.09 (0.91–1.31)	0.86 (0.61–1.23)
Rural	1.12 (1.00–1.24)*	1.42(1.11–1.83)**	1.13 (0.99–1.30)	1.22 (0.87–1.72)	1.10 (0.94–1.29)	1.32 (0.93–1.88)

***p < 0.001 ** *p* < 0.01 *p < 0.05 Baseline *BMI* > 30 kg/m^2^ Body Mass Index, *AOR* Adjusted Odds Ratio

^a^ Adjusted for age, sex, race, ethnicity, region, income, rural/urban, registration date and program

^b^ Adjusted for age, sex, race, ethnicity, baseline BMI category, region, income, rural/urban, registration date and program

^c^ Adjusted for age, sex, race, ethnicity, baseline BMI category, region, income, rural/urban, registration date, program weeks attended and program

^d^Engaged measured by platform: online: composite of curriculum delivery, health coach interaction, peer support and self-tracking that is measured equivalent of in-person hour attendance and agreed upon between the payer and program; In-person: physical attendance of hour long weekly session

^e^ Includes Multiracial and Not Reported. Hawaiian/Pacific Islander not reported given small sample size

^f^Household income estimates based on participant zip codes using Zip Code Tabulation Area median household incomes from the US Census Data

^g^Rurality estimates based on participant zip codes using the Rural–Urban Commuting Area Codes (RUCA) which uses 2010 census data on the basis of population density, urbanization, and daily commuting pattern

### Reach

A higher proportion of adults matched to online programs enrolled, compared with adults referred to in-person programs (Table 2; 56% vs 51%, *p* < 0.001). After adjusting for age, sex, race/ethnicity, region, income, rural/urban, registration date and program, the odds of an online participant enrolling after being referred (i.e. reach) was twice as high among those 18–44 years old than among those ages greater than 65, and 55% higher among those 45–64 years old than among those ages 65 and older (*p* < 0.001). However, age was not significantly associated with increased reach among in-person participants. Reach was higher in rural participants (compared to urban participants) for both online (*p* < 0.05) and in-person delivery (*p* < 0.05).

### Program engagement

Over half (55%) of enrolled online participants remained in the program after 1 year, compared to about a quarter (28%) of enrolled in-person participants (*p* < 0.001). Engagement at 6-months (engagement with ≥9 sessions by week 26) was significantly lower among online-enrolled participants compared to in-person (46% vs 66%, *p* < 0.001). After adjusting for age, sex, race/ethnicity, baseline BMI, region, income, rural/urban, registration date and program, the odds of an online participant engaged at 6-months was lower among those 18–44 years old (AOR 0.39,95%CI 0.33–0.45) and 45–64 years old (AOR 0.72,95%CI 0.62–0.84) than among those ages 65 and older (Table 3). Similarly, 6-month program engagement among online participants was lower among participants with baseline obesity compared to those who were overweight (*p* < 0.01). After adjusting for age, sex, race/ethnicity, baseline BMI, region, income, rural/urban, registration date and program, the odds of participants achieving meaningful weight loss was significantly higher for each session engaged (in-person - AOR 1.08, 95%CI 1.07–1.10; online 1.04, 95%CI 1.03–1.04, see Additional file 3). Adjusting for engagement partially attenuated the effectiveness of in-person programs, suggesting factors other than engagement may be driving the differential effectiveness of the in-person format (see Additional file 5).

### Sensitivity analyses

There was a similar pattern of effectiveness (5% weight loss) by format-type among participants who engaged in at least four sessions (28% online compared to 41% in-person, *p* < 0.001). The difference in effectiveness by format was only partially attenuated when defining percent weight loss as the difference between baseline and lowest measured follow-up weight (38% online compared to 45% in-person among enrolled participants, *p* < 0.001). Excluding participants with multiple participation records did not modify patterns. None of the sensitivity analyses substantively impacted results overall or by subgroups (Supplemental material; Additional file 3).

## Discussion

In this prospective analysis of a multistate referral registry of LCP to prevent diabetes, we found that adults referred to online LCP were more likely to enroll but less likely to stay engaged compared to adults referred to in-person programs. There was modest clinically meaningful weight loss among adults enrolled in online programs across all levels of engagement. This trade-off—higher reach and lower effectiveness—has key implications for diabetes prevention at a national scale.

To our knowledge, this is the first estimate of meaningful weight loss among a large sample of adults enrolled across in-person and online platforms. The weight loss estimate observed in online programs was modest in comparison to both the in-person LCP estimates in this sample and previous large-scale in-person estimates (35 > 5% weight loss [22], mean percent weight loss 4.8–5.2% [22, 23]). Although meaningful weight loss differed significantly by specific online provider in this study, outcomes were consistently lower than in-person estimates. Furthermore, mean weight loss estimates were lower than recent Veteran Administration (3.7% [10]) and industry-supported (4.7, 7.5% [11, 13]) studies of online LCP. These findings raise uncertainty about the comparability of health benefits between delivery formats in the real-world setting [24].

Hypothetical consideration of national adoption of exclusively online-supported LCP can help contextualize the advantages and disadvantages of this programming [25]. Applying the reach and effectiveness trade-offs in this study and the same eligibility criteria, exclusive online LCP may result in roughly 18% fewer incident cases of diabetes prevented over 10 years than accessible in-person programming among insured adults, given multiple assumptions.^2^

Commercially insured, at-risk adults may have a choice in platform in up to 23% of counties in the US as of early 2017 [26]. In areas without any current LCP access, online LCP offers an appealing alternative to in-person LCP in scalability. In settings where both formats are accessible, numerous questions persist as to how the current delivery model will impact population diabetes prevention. It remains unclear if multi-format delivery supports incremental population benefit (e.g. participants who achieved meaningful weight loss in online LCP would not have benefited from in-person and/or other formats such as telehealth [27] LCP alone). Online referral was higher among difficult to reach subgroups (younger, rural, less obese and male) and may suggest broader reach with online programming. Assuming online LCP does improve incremental population health, an economic evaluation could determine if this benefit outweighs the costs of integrating and maintaining a national multi-platform system delivery model. Lastly, it is unlikely that the current landscape offers program availability that matches eligible adults’ preferences, especially among targeted risk groups. Further examination of participant behavior may help inform policy-makers how to optimize the large-scale implementation of DPP to align with demand and preference.

In this study, we found a dose-trend relationship between engagement and median weight loss among online participants. The dose trend was similar to the in-person National Diabetes Prevention Program (NDPP) evaluation [22], but lower than the CDC-defined weight loss threshold of 5% across all levels of engagement. This finding may reflect both the relatively passive nature of online engagement (e.g. home weigh-ins) as well as the challenge of defining generalizable engagement measures across digital programs. Future studies would benefit from standardization of online program delivery measures to identify the appropriate digital engagement targets to support a performance-based delivery model for online LCP.

Over half of adults enrolled in online LCP continued to weigh-in after 12 months, consistent with high passive retention rates found in a previous evaluation of a single online program [11]. However, our estimate of meaningful engagement at 6 months (46% > 9 sessions) was lower than the Veteran’s Administration’s evaluation of a single online LCP (86.7% > 8 sessions) [10]. As Grock et al. describes, “While the accessibility of digital technology allows users to initiate interventions effortlessly, it also allows users to disengage easily [28].” Relatively low yet persistent levels of engagement among online users creates potential opportunities for online LCP to activate individuals and increase the proportion reaching the successful weight loss threshold.

### Limitations

Our study has several important limitations. Because many participants selected their programming, propensity to engage was likely higher than in research studies. Further, we do not have data on the participants that were offered a choice of in-person versus online programming. While this limits our ability to weigh the contribution of selection bias and examine the role choice may have on engagement, this analysis reflects the real-world, coinciding delivery of online and in-person platforms where only a minority have access to in-person programming. Program differences may be attenuated since we include a small sample of participants that participated in both online and in-person programs. We present the unadjusted proportions to demonstrate the impact of LCP among high-risk adults in a large, real-world setting. We used separate models within each platform (in-person and online) to optimize model fitness and differences in measure definitions (e.g. ascertainment of weight, engagement). These unadjusted comparisons may be biased given the participant and setting level differences between the two platforms (see Table 1). However, our findings remain similar when adjusting for participant and setting difference between the two platforms in a combined analysis (see Additional file 4).

There was a high degree of missingness in our data, particularly among in-person participants. This is likely partially related to differences in ascertainment of weight (in-person weigh-in during sessions vs. online cellular scale). To address this limitation, we designed our study to reflect national program benchmarks that reflect real-world metrics that inform policy. Further, we tested multiple assumptions of missingness in our sensitivity analyses. The difference in missingness raises the uncertainty of effectiveness and estimated weight loss among in-person participants. We caution readers that these findings reflect real-world delivery of LCP and do not reflect a comparative effectiveness trial of online and in-person lifestyle change programs within a controlled setting. Since this dataset was fully deidentified, some of our participant-level variables are area estimates (income, rurality). Finally, the CDC-recognized LCP were limited to Solera’s referral network among commercially-insured adults and may not generalize to NDPP as a whole.

This study also has several strengths. This is a large, real-world dataset of online-LCP participants. Our in-person results align with the CDC evaluation, suggesting our findings may be generalizable to national estimates of commercially-insured adults participating in DPRP-recognized organizations.

## Conclusion

Expanding accessibility of online delivery of LCP is an attractive strategy to bring DPP to scale. Modest achievement of meaningful weight loss in online programs temper the potential population impact in diabetes prevention. Further examination of comparative effectiveness, barriers and enablers of scalability and participant preference will better inform how to expand and optimize national LCP multi-format delivery.

## Supplementary Information

**Additional file 1.** Operational Map of Online Referral Registry. Operational map of the Solera online registry with evaluation definitions.

**Additional file 2.** Statistical Supplement. Description of model components used to estimate per week weight loss.

**Additional file 3.** Results Supplement. Table of Sensitivity Model Outputs.

**Additional file 4.** Results Supplement. Table of Adjusted Implementation Outcomes from Combined Models.

**Additional file 5.** Results Supplement. Table of Adjusted Effectiveness by Class Engaged from Combined Models.

## Data Availability

Data was derived from the program registry; no questionnaire was developed for this study. The dataset generated and analyzed for the current study is not publicly available since it is data managed by Solera Health. Data may be available on reasonable request to Solera Health.

## Abbreviations

T2D: Type 2 diabetes
US: United States
LCP: Lifestyle change programs
CDC: Center for Disease Control and Prevention
DPP: Diabetes prevention program
NDPP: National Diabetes Prevention Program
DPRP: Diabetes Prevention Recognition Program
BMI: Body mass index
ZCTA: Zip code tabulation areas
RUCA: Rural-urban commuting area codes

## Footnotes

1 For simplicity, hereafter we refer to all programming based on Diabetes Prevention Program (DPP) services as Lifestyle Change Programs (LCP), including when referring to DPP implemented prior to the CDC’s 2018 change in terminology from DPP to LCP.

2 Cited In-person LCP estimates assuming risk-stratified referral approach: Ackermann et al.: 36 million linked to LCP. ~ 33% referred will attend: ~ 12 million enroll in LCP * 0.184 rate of DM over 10 yrs. * 0.58 relative risk reduction among > 5% weight loss * 0.35 achieve > 5% weight loss in in-person = ~ 448,500 cases avoided Adopting online LCP (5% increase in attendance among referred and 10% decrease in effectiveness among referred compared to in-person): 36 million linked to LCP. ~ 38% referred will attend: ~ 13.8 million enroll in LCP * 0.184 rate of DM over 10 yrs. * 0.58 relative risk reduction among > 5% weight loss * 0.25 achieve > 5% weight loss in in-person = ~ 368,025 cases avoided
