# Supplementary material for: Reach, engagement and effectiveness of in-person and online lifestyle change programs to prevent diabetes
Source: BMC Public Health. 2021 Jul 5;21:1314. doi: 10.1186/s12889-021-11378-4 (PMC8256225; doi:10.1186/s12889-021-11378-4)
Supplement: Supplementary file 1 — Additional file 1. Operational Map of Online Referral Registry. Operational map of the Solera online registry with evaluation definitions. [file 12889_2021_11378_MOESM1_ESM.docx]

Figure S1: Operational Map of Online Referral Registry (*Solera4me.com*) to Lifestyle Change Programs to Prevent Diabetes^a,b^


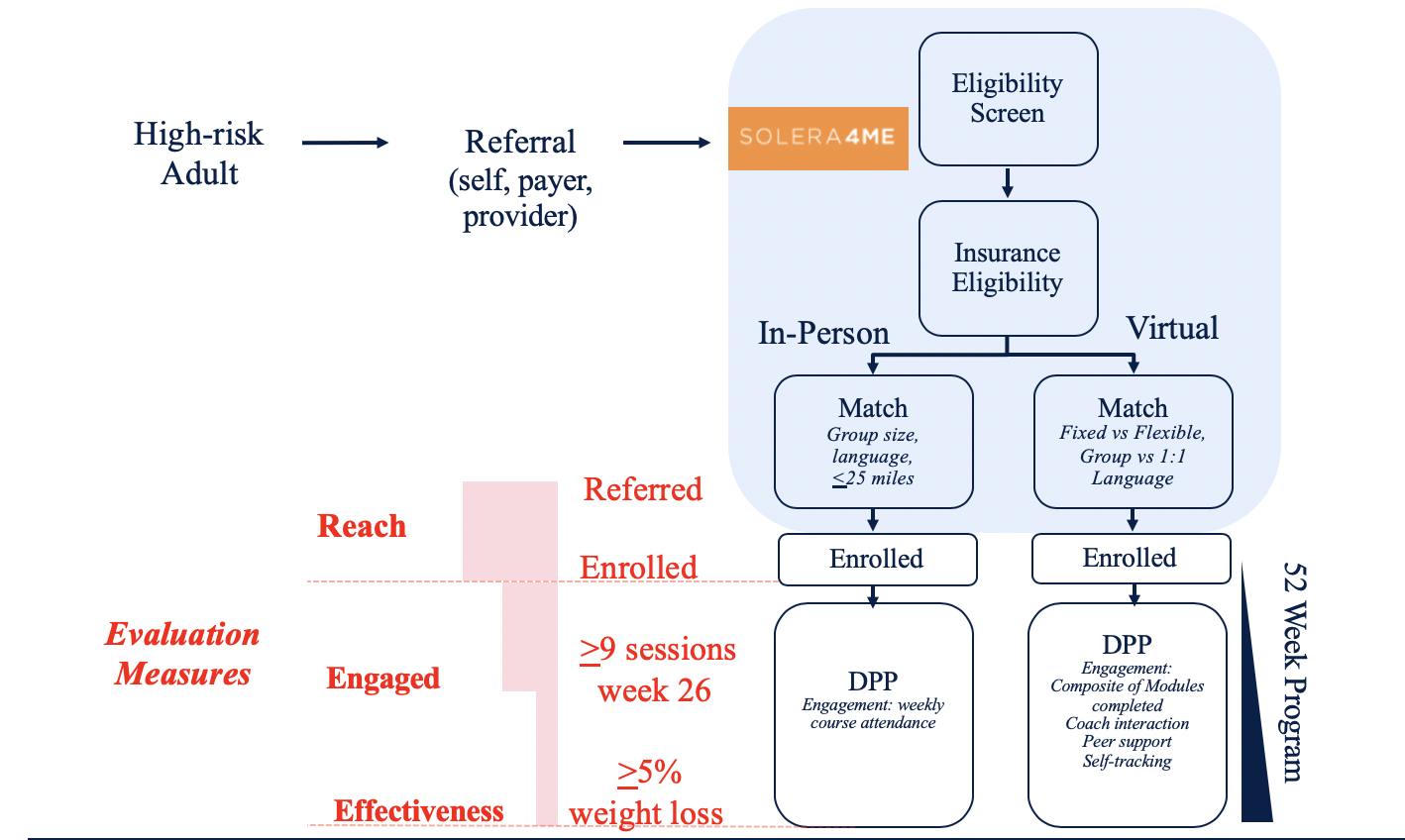


^d^

^c^

^a^Multilevel referral network connecting referral organizations (commercial insurers, federal qualified health centers, integrated health systems) with LCP organizations (in-person and online platforms), leveraging system support (National Diabetes Prevention Program).

^b^Operational differences:

Enrollment: In-person: live coordinator contacts participant; Online: Email instructions to complete app download and registration

Weigh-in: In-person: weigh-in by health coach in class; Online: digital scale at home

Content delivery: In-person: weekly classes including content delivery, peer-support exercise, self-tracking support, health coach interaction; Online: On-demand curriculum modules, forum and group chat peer support, 1:1 live health coach interaction by phone and/or chat, app-based nutrition and/or activity tracking

^c^Referral activities included Insurer: audit of members and referral instruction letters to providers and/or individual deemed high-risk; Care organizations- letters to eligible patients with online referral instructions, integration with system DPP activities; Self: Direct consumer marketing

^d^Each online program defined engagement as a composite of subcomponents equivalent to an in-person hour session. Subcomponents included curriculum delivery, health coach interaction, peer support and self-tracking (physical and/or dietary). Each program had a unique threshold for engagement deemed equivalent to an hour of in-person class, defined between the program and commercial insurer for reimbursement.

Figure S2: Monthly Retention^a^ by weigh-in among Participants Enrolled in a Community Lifestyle Change Programs to Prevent Diabetes (n=10,906 Online and 3,243 In-person Enrolled)

^Online^

p<0.001^b^

^a^Monthly program retention was estimated by the proportion of enrolled participants with at least one weight measure (online: home weight recorded by cellular scale; in-person: weight measured by health coach at class) and program contact (online: any login recorded; in-person: class attendance).

^b^ p values estimated using logrank test
