# Supplementary material for: Reach, engagement and effectiveness of in-person and online lifestyle change programs to prevent diabetes
Source: BMC Public Health. 2021 Jul 5;21:1314. doi: 10.1186/s12889-021-11378-4 (PMC8256225; doi:10.1186/s12889-021-11378-4)
Supplement: Supplementary file 2 — Additional file 2. Statistical Supplement. Description of model components used to estimate per week weight loss. [file 12889_2021_11378_MOESM2_ESM.docx]

**Statistical Supplement**

*Model Components*

We used multilevel mixed-effects modelling with participants nested within the program as random effect and exchangeable correlation, adjusted for participant- and setting-level factors, to estimate the trajectory in weight loss. Since missingness and program participation differed significantly by platform type, we developed separate models for virtual and in-person programs. We included participant-level random intercept and slope and program-level random intercept into the online models and only participant-level random intercept and slope for in-person since most participants were clustered within one program.

We incorporated potential confounders (age, gender, ethnicity, registration date and state) based on our a priori conceptual framework, existing bivariate associations and prior literature. We then used stepwise change in coefficient (>10% threshold) to decide whether to incorporate additional covariates (Zip code-derived household income met threshold; rural designation did not meet threshold). We then included significant time-variant interaction from bivariate models to the final model (age x time, ethnicity x time). Model diagnostics included assessment for collinearity, normality and constant variance of residuals and heteroskedasticity. We assumed missing at random (MAR) and used multiple imputations (N=20) with chained imputations for our in-person estimates (estimates using multiple imputations did not significantly differ in our online models).

*Sensitivity*

Given that weekly weight loss may be a result of program ‘dosage’ up to the given week, we examined weight loss by cumulative program attendance. To examine the impact of nonlinear trend of weight loss (weight slope plateau around week 26), we conducted two-piece linear (pre- and post- week 26) and quadratic models. Models using cumulative program attendance and two-piece linear differed significantly in our primary model, reflecting a larger rate of weight loss within the first 6-months of the program. The one-year model estimates however, did not modify our linear model outputs. One-year model weight-loss estimates from the quadratic models were significantly lower than our linear models (~20-30%) but did not alter our study’s significant findings. For interpretability and generalizability to similar large analysis, we decided to report our linear model findings. To examine the robustness of the conclusions to the MAR assumption, we examined our model estimates by populating missing weights by a) <4-week mean weight trend; b) most recent weight in past month and c) estimate based on total measured program weight linear model. The model estimate of weekly weight loss did not differ significantly when we populated missing weights by the aforementioned assumptions.

**Missing Weights by Month among Participants Enrolled in Community Lifestyle Change Program to Prevent Diabetes in a Multistate Referral Registry (2015-2018)**

| **Month** | **In-person**  **n=3,243** | **Online**  **n=10,906** |
| --- | --- | --- |
| **1** | 2 (0%) | 13 (0.1%) |
| **2** | 380 (12%) | 877 (8%) |
| **3** | 605 (19%) | 1,478 (14%) |
| **4** | 866 (27%) | 2,089 (19%) |
| **5** | 1,040 (32%) | 2,552 (23%) |
| **6** | 1,190 (37%) | 2,919 (27%) |
| **7** | 1,378 (42%) | 3,285 (30%) |
| **8** | 1,507 (46%) | 3,581 (33%) |
| **9** | 1,654 (51%) | 3,889 (36%) |
| **10** | 1,830 (56%) | 4,226 (39%) |
| **11** | 2,005 (62%) | 4,520 (42%) |
| **12** | 2,335 (72%) | 4,936 (45%) |
