# Supplementary material for: Reach, engagement and effectiveness of in-person and online lifestyle change programs to prevent diabetes
Source: BMC Public Health. 2021 Jul 5;21:1314. doi: 10.1186/s12889-021-11378-4 (PMC8256225; doi:10.1186/s12889-021-11378-4)
Supplement: Supplementary file 3 — Additional file 3. Results Supplement. Table of Sensitivity Model Outputs. [file 12889_2021_11378_MOESM3_ESM.docx]

Adjusted Odd Ratios of Reach, Engagement and Effectiveness by Participant- and Setting-level characteristics in Community Lifestyle Change Programs to Prevent Diabetes in a Multistate Referral Registry (2015-2018) from the Sensitivity Analyses

|  | **Effectiveness**  >5% weight loss/>4 sessions engaged^d^ | |  | **Effectiveness**  >5% weight loss by lowest weight/enrolled | |  | **Effectiveness**  >5% weight loss/Enrolled (exclude multiple) | |  |
| --- | --- | --- | --- | --- | --- | --- | --- | --- | --- |
|  | **Online**  n=2,123/7,591  AOR^a^ (95%CI) | **In-Person**  n=1,119/2,762  AOR^a^ (95%CI) |  | **Online**  n=4,148/10,906  AOR^b^ (95%CI) | **In-Person**  n=1,463/3,243  AOR^b^ (95%CI) |  | **Online**  n=8,312/10,819  AOR^c^ (95%CI) | **In-Person**  n=1,101/3,166  AOR^c^ (95%CI) | |
| **Participant** |  |  |  |  |  |  |  |  | |
| Age (ref >65) |  |  |  |  |  |  |  |  | |
| 18-44 | 0.61 (0.50-0.75)*** | 0.75 (0.56-0.99)* |  | 0.77 (0.65-0.91)** | 0.79 (0.59-1.06) |  | 0.67 (0.56-0.80)*** | 0.74 (0.56-0.99)* | |
| 45-64 | 0.74 (0.62-0.89)** | 0.67 (0.52-0.86)*** |  | 0.90 (0.77-1.05) | 0.68 (0.53-0.88)** |  | 0.81 (0.69-0.95)** | 0.65 (0.51-0.83)** | |
| Sex (ref male) | 0.87 (0.78-0.99)* | 0.62 (0.48-0.80)*** |  | 0.96 (0.87-1.06) | 0.56 (0.43-0.73)*** |  | 0.95 (0.85-1.05) | 0.62 (0.48-0.80)*** | |
| Race (ref white) |  |  |  |  |  |  |  |  | |
| African American | 0.74 (0.59-0.99)* | 0.58 (0.40-0.86)** |  | 0.75 (0.63-0.91)* | 0.51(0.36-0.74)** |  | 0.81 (0.66-1.00)* | 0.60 (0.41-0.88)** | |
| Asian American | 0.93 (0.71-1.11) | 0.35 (0.20-1.03) |  | 0.97 (0.77-1.23) | 0.66 (0.32-1.39) |  | 0.94 (0.71-1.23) | 0.48 (0.21-1.09) | |
| Native American | 0.61 (0.31-1.20) | 1.00 (0.31-3.32) |  | 1.11 (0.69-1.78) | 0.88 (0.25-3.03) |  | 0.71 (0.40-1.26) | 1.08 (0.32-3.64) | |
| Other^e^ | 0.84 (0.71-0.99)* | 1.02 (0.80-1.31) |  | 1.00 (0.87-1.14) | 1.07 (0.84-1.37) |  | 0.90 (0.78-1.05) | 1.04 (0.81-1.33) | |
| Ethnicity (ref non-Latino)^e^ |  |  |  |  |  |  |  |  | |
| Latino | 0.91 (0.73-1.12) | 0.87 (0.64-1.19) |  | 0.91 (0.77-1.08) | 0.92 (0.68-1.25) |  | 0.92 (0.76-1.11) | 0.87 (0.63-1.19) | |
| BMI (ref Overweight) |  |  |  |  |  |  |  |  | |
| Obese | 0.98 (0.88-1.10) | 1.10 (0.92-1.33) |  | 0.94 (0.86-1.03) | 1.07 (0.89-1.28) |  | 1.00 (0.91-1.11) | 1.12 (0.93-1.34) | |
| **Setting** |  |  |  |  |  |  |  |  | |
| Region (Ref California) |  |  |  |  |  |  |  |  | |
| Florida | 1.06 (0.93-1.22) | 1.14 (0.93-1.40) |  | 1.02 (0.92-1.23) | 1.20 (0.98-1.47) |  | 1.08 (.095-1.21) | 1.18 (0.96-1.44) | |
| Colorado | 0.71 (0.51-0.98)* | 1.07 (0.78-1.49) |  | 0.76 (0.58-0.99*) | 0.99 (0.72-1.36) |  | 0.77 (0.57-1.05) | 1.13 (0.81-1.56) | |
| Other | 1.15 (0.95-1.39) | 0.84 (0.49-1.46) |  | 0.98 (0.84-1.14) | 0.66 (0.38-1.15) |  | 1.18 (1.00-1.41)* | 0.80 (0.45-1.39) | |
| Income (ref: highest)^f^ |  |  |  |  |  |  |  |  | |
| <40,000 | 1.05 (0.88-1.26) | 1.12 (0.85-1.49) |  | 1.06 (0.92-1.23) | 1.13 (0.85-1.49) |  | 1.07 (0.91-1.25) | 1.09 (0.83-1.44) | |
| 40,001-50,000 | 1.06 (0.92-1.23) | 0.93 (0.75-1.17) |  | 1.07 (0.95-1.20) | 0.95 (0.76-1.18) |  | 1.03 (0.91-1.17) | 0.91 (0.73-1.15) | |
| 50,001-63,000 | 0.95 (0.82-1.09) | 0.91 (0.74-1.12) |  | 0.99 (0.89-1.11) | 0.91 (0.74-1.11) |  | 0.97 (0.86-1.10) | 0.89 (0.73-1.10) | |
| Rurality (ref Urban)^g^ |  |  |  |  |  |  |  |  | |
| Suburban | 1.14 (0.93-1.40) | 0.86 (0.61-1.23) |  | 1.05 (0.89-1.24) | 0.77 (0.54-1.09) |  | 1.10 (0.91-1.32) | 0.88 (0.62-1.25) | |
| Rural | 1.05 (0.88-1.25) | 1.29 (0.91-1.84) |  | 1.03 (0.89-1.19) | 1.23 (0.86-1.76) |  | 1.11 (0.95-1.30) | 1.35 (0.94-1.93) | |

***p<0.001 ** p<0.01 *p<0.05 Baseline BMI: >30 kg/m^2^ Body Mass Index AOR: Adjusted Odds Ratio

^a^ Adjusted for age, sex, race, ethnicity, region, income, rural/urban, registration date and program

^b^ Adjusted for age, sex, race, ethnicity, baseline BMI category, region, income, rural/urban, registration date and program

^c^ Adjusted for age, sex, race, ethnicity, baseline BMI category, region, income, rural/urban, registration date, program weeks attended and program

^d^Engaged measured by platform: online: composite of curriculum delivery, health coach interaction, peer support and self-tracking that is measured equivalent of in-person hour attendance and agreed upon between the payer and program; In-person: physical attendance of hour long weekly session

^e^ Includes Multiracial and Not Reported. Hawaiian/Pacific Islander not reported given small sample size.

^f^Household income estimates based on participant zip codes using Zip Code Tabulation Area median household incomes from the US Census Data.

^g^Rurality estimates based on participant zip codes using the Rural–Urban Commuting Area Codes (RUCA) which uses 2010 census data on the basis of population density, urbanization, and daily commuting patterns.

Adjusted Odd Ratios of Reach, Engagement and Effectiveness by Participant- and Setting-level characteristics in Community Lifestyle Change Programs to Prevent Diabetes in a Multistate Referral Registry (2015-2018) from the Sensitivity Analyses

|  | **Effectiveness**  >5% weight loss/Enrolled | |  |
| --- | --- | --- | --- |
|  | **Online**  n=4,148/10,906  AOR^a^ (95%CI) | **In-Person**  n=1,463/3,243  AOR^a^ (95%CI) |  |
| **Engagement** |  |  |  |
| Sessions Engaged^b^ | 1.04 (1.03-1.04)*** | 1.08 (1.07-1.10)*** |  |
| **Participant** |  |  |  |
| Age (ref >65) |  |  |  |
| 18-44 | 0.84 (0.70-1.01) | 0.84 (0.62-1.13) |  |
| 45-64 | 0.87 (0.74-1.03) | 0.68 (0.53-0.88)*** |  |
| Sex (ref male) | 0.93 (0.84-1.03) | 0.61 (0.47-0.79)*** |  |
| Race (ref white) |  |  |  |
| African American | 0.80 (0.65-0.99)* | 0.63 (0.43-0.94)** |  |
| Asian American | 0.96 (0.73-1.28) | 0.51 (0.21-1.21) |  |
| Native American | 0.77 (0.43-1.38) | 1.31 (0.36-4.63) |  |
| Other^c^ | 0.91 (0.78-1.06) | 1.02 (0.79-1.31) |  |
| Ethnicity (ref non-Latino) |  |  |  |
| Latino | 0.96 (0.79-1.16) | 0.87 (0.64-1.19) |  |
| BMI (ref Overweight) |  |  |  |
| Obese | 1.01 90.91-1.12) | 0.90 (0.65-1.25) |  |
| **Setting** |  |  |  |
| Region (Ref California) |  |  |  |
| Florida | 1.18 (0.98-1.25) | 1.20 (0.97-1.48) |  |
| Colorado | 0.77 (0.57-1.04) | 1.06 (0.76-1.48) |  |
| Other | 1.19 (0.99-1.41) | 0.78 (0.44-1.39) |  |
| Income (ref: highest)^d^ |  |  |  |
| <40,000 | 1.09 (0.93-1.28) | 1.10 (0.83-1.47) |  |
| 40,001-50,000 | 1.06 (0.93-1.21) | 0.93 (0.74-1.18) |  |
| 50,001-63,000 | 0.97 (0.86-1.10) | 0.90 (0.73-1.11) |  |
| Rurality (ref Urban)^e^ |  |  |  |
| Suburban | 1.06 (0.88-1.28) | 0.96 (0.65-1.37) |  |
| Rural | 1.07 (0.91-1.26) | 1.48 (1.03-2.11) |  |

***p<0.001 ** p<0.01 *p<0.05 Baseline BMI: >30 kg/m^2^ Body Mass Index AOR: Adjusted Odds Ratio

^a^ Adjusted for age, sex, race, ethnicity, baseline BMI category, region, income, rural/urban, registration date, program weeks attended and program

^b^Engaged measured by platform: online: composite of curriculum delivery, health coach interaction, peer support and self-tracking that is measured equivalent of in-person hour attendance and agreed upon between the payer and program; In-person: physical attendance of hour long weekly session

^c^ Includes Multiracial and Not Reported. Hawaiian/Pacific Islander not reported given small sample size.

^d^Household income estimates based on participant zip codes using Zip Code Tabulation Area median household incomes from the US Census Data.

^e^Rurality estimates based on participant zip codes using the Rural–Urban Commuting Area Codes (RUCA) which uses 2010 census data on the basis of population density, urbanization, and daily commuting patterns.
