# Supplementary material for: Reach, engagement and effectiveness of in-person and online lifestyle change programs to prevent diabetes
Source: BMC Public Health. 2021 Jul 5;21:1314. doi: 10.1186/s12889-021-11378-4 (PMC8256225; doi:10.1186/s12889-021-11378-4)
Supplement: Supplementary file 4 — Additional file 4. Results Supplement. Table of Adjusted Implementation Outcomes from Combined Models. [file 12889_2021_11378_MOESM4_ESM.docx]

Implementation Outcomes in Combined Models^a^ of Community Lifestyle Change Program to Prevent Diabetes in a Multistate Referral Registry (2015-2018)

| **Implementation Measures** | | **Predicted Proportion**^b^  (95% CI) | |
| --- | --- | --- | --- |
|  | **Online** | | **In-Person** |
| Referred^c^ |  | |  |
| **Reach**  *(Enrolled/Referred)* | 50.4% (49.2- 51.7) | | 56.4% (55.7-57.1) |
| Enrolled |  | |  |
| **Engagement**  *(>9 weeks attended*^d^ *by week 26 / Enrolled)* | 45.4% (4.54-47.3) | | 63.3% (61.6-65.0) |
| **Effectiveness**^e^  *(>5% weight loss/Enrolled)* | 22.4% (21.6-23.2) | | 38.3% (36.6-40.0) |

Baseline BMI: >30 kg/m^2^ Body Mass Index

^a^Our primary analyses used separate models within each platform (in-person and online) to optimize model fitness and differences in measure definitions (e.g. ascertainment of weight, engagement). We present a sensitivity analysis here using combined (in-person and online) models to adjust for participant and program level differences between the two platforms for comparison.

^b^Predicted using postregression margins command in Stata. Adjusted for age, sex, race, ethnicity, region, income, rural/urban, registration date, platform type (+ baseline BMI category for ‘Engagement’ and + baseline BMI category and program weeks attended for ‘Effectiveness’)

^c^Participants were considered referred after they registered and matched to a lifestyle change program per their preferences. Participants that lived more than 25 miles from closest in-person program were limited to online programs.

^d^Attendance measured by platform: online: composite of curriculum delivery, health coach interaction, peer support and self-tracking that is measured equivalent of in-person hour attendance and agreed upon between the payer and program; In-person: physical attendance of hour long weekly session

^e^% weight loss calculated by % change from baseline to last recorded weight
