# Supplementary material for: Reach, engagement and effectiveness of in-person and online lifestyle change programs to prevent diabetes
Source: BMC Public Health. 2021 Jul 5;21:1314. doi: 10.1186/s12889-021-11378-4 (PMC8256225; doi:10.1186/s12889-021-11378-4)
Supplement: Supplementary file 5 — Additional file 5. Results Supplement. Table of Adjusted Effectiveness by Class Engaged from Combined Models. [file 12889_2021_11378_MOESM5_ESM.docx]

Effectiveness of Online Programs in Combined Models^a^ of Community Lifestyle Change Program to Prevent Diabetes in a Multistate Referral Registry (2015-2018)

| **Implementation Measure** | | AOR  (ref in-person) |
| --- | --- | --- |
| **Effectiveness**^b^  *(>5% weight loss/Enrolled)* | 0.43  (0.40-0.48)*** | |
| **Effectiveness** *+ adjusted for engagement) ^c^* | 0.64  (0.58-0.71)*** | |

***p<0.001 ** p<0.01 *p<0.05 AOR: Adjusted Odds Ratio

% weight loss calculated by % change from baseline to last recorded weight

^a^Our primary analyses used separate models within each platform (in-person and online) to optimize model fitness and differences in measure definitions (e.g. ascertainment of weight, engagement). We present a sensitivity analysis here using combined (in-person and online) models to adjust for participant and program level differences between the two platforms for comparison.

^b^Adjusted for age, sex, race, ethnicity, region, income, rural/urban, registration date, baseline BMI category and program weeks attended

^c^Adjusted for age, sex, race, ethnicity, region, income, rural/urban, registration date, baseline BMI category and program weeks attended + total classes engaged. In-person engagement was defined as physical attendance at the weekly hour-long session tallied by the health coach. Weekly online engagement was defined by a proprietary algorithm which includes curriculum delivery, health coach interaction, peer support and self-tracking that is equivalent to an hour of in-person class engagement.
